# Evaluation and analysis of the projected population of China

**DOI:** 10.1038/s41598-022-07646-x

**Published:** 2022-03-07

**Authors:** Kaixuan Dai, Shi Shen, Changxiu Cheng

**Affiliations:** 1 State Key Laboratory of Earth Surface Processes and Resource Ecology, Beijing Normal University, Beijing, 100875 China; 2 Key Laboratory of Environmental Change and Natural Disaster, Beijing Normal University, Beijing, 100875 China; 3 Faculty of Geographical Science, Beijing Normal University, Beijing, 100875 China; 4 National Tibetan Plateau Data Centers, Beijing Normal University, Beijing, 100101 China

**Keywords:** Climate-change policy, Socioeconomic scenarios, Sustainability

## Abstract

The population has a significant influence on economic growth, energy consumption, and climate change. Many scholars and organizations have published projections for China's future population due to its substantial population amounts. However, these projections have not been evaluated or analyzed, which may lead confusion to extensional studies based on these datasets. This manuscript compares several China's projection datasets at multiscale and analyzes the impacting factors affecting projection accuracy. The results indicate that the slow of actual population growth rates from 2017 is earlier than most datasets projected. Therefore, the turning point of population decline probably comes rapidly before these datasets expected during 2024 and 2034. Furthermore, the projections do not reveal the population decline from 2010 in the Northeast provinces such as Jilin and Heilongjiang, and underrate the population increase in the southern provinces such as Guangdong and Chongqing. According to the results of regression models, the rate of population changes and the number of migrations people play a significant role in projection accuracy. These findings provide meaningful guidance for scholars to understand the uncertainty of those projection datasets. Moreover, for researchers performing population projections, our discoveries provide insights to increase the projection accuracy.

## Introduction

At present, human activities have become the dominant force in Earth's ecological processes and global climate change, which indicates Earth has entered a new epoch, Anthropocene^1,2^. The highly intensive human activities have caused global temperature to warm by approximately 1.09 °C since the industrial revolution in the 1700s^3^. According to China's seventh national population census, the total population was 1.41 billion at the end of 2020, accounting for about 18% of the global population^4^.

As the largest populated and most active economic development country, China's vast population provides a large consumer market with more business opportunities for enterprises^5–8^. However, overpopulation negatively influences natural resources, the ecological environment, and global climate change^9,10^. Moreover, the growing population has a critical influence on achieving the Sustainable Development Goals (SDG), such as urban expansion control (SDG 11.3.1) and education equality (SDG 4.6.1)^11^. Therefore, China's future population growth is a crucial issue that has attracted international attention.

Many international organizations have estimated China's future population without spatial properties. For instance, the World Bank has estimated national total populations and age compositions with different economic development levels until 2050^12^. The United Nations (UN) has assessed previous global population growth situations and projected future world populations in prospect reports^13^. The International Institute for Applied Systems Analysis (IIASA) has provided a country-scale projection population dataset under different shared socioeconomic pathways (SSPs) from 2010 to 2100^14^. These national-scale projections could reveal the general population growth tendency and serve as inputs for addressing natural and socioeconomic issues. For example, Scovronick et al. analyzed the impact of population growth on world climate change policies based on the UN future population projection dataset^15^. Dottori et al. explored the threat of river flooding based on IIASA population projections under different anthropogenic warming scenarios^16^. Li et al. used the IIASA's SSP population and GDP projection data to forecast worldwide urban expansion conditions^17^. However, the national data cannot reflect the spatial heterogeneity of population distribution and is insufficient to support policy decision-making at local scales.

As a result, several researchers have created spatially explicit population projections at a small scale. For example, Jones and O'Neill projected global population values from 2000 to 2100 over 5 years under 5 SSP scenarios^18^. Gao converted the global 1/8-degree grid data of Jones and O'Neill to 1-km degree grids by constructing a downscaling transform weight matrix, thereby providing more accurate and detailed data in small regions^19^. Furthermore, the Japanese National Institute for Environmental Studies (NIES) created global population projection datasets from 1980 to 2100 over 10 years with 0.5-degree grids, although these datasets included only the SSP1, SSP2, and SSP3 scenarios^20^. To accurately grasp China's future population growth tendency, the projections of NUIST (Nanjing University of Information Science and Technology) and THU (Tsinghua University) were created recently by Huang et al.^21^ and Chen et al.^22^, respectively. These spatially explicit population datasets have been widely used to explore the influence of future population levels on global climate change^23–26^, extreme weather disaster events^27–29^, land-use change^24^, and ecosystem service change^30^. Although they have been applied in many research fields, we know little about their projection accuracy and poorly understand the factors that affect their projection accuracy. Moreover, uncertainties remain about these datasets, which have hindered further investigations and research on adaptations to climate change and sustainability. Consequently, it is necessary to evaluate their projection accuracy and determine their applicability in different regions.

This study compares China's population projections with actual census data from 2010 to 2020 at different scales. Then, the spatial error regression model (SEM) is applied to attribute the factors affecting projection accuracy. The contributions of this study are evaluating the gaps between actual and projection populations and analyzing the contributions of various impact factors to projection accuracy. In addition, the results provide a better understanding of China's population growth situations and the characteristics of different projection datasets. Besides, it also provides insights into the parameters adjustment of projection models to reduce the projection errors in the future^31,32^.

The rest of this paper is organized as follows. Second section presents the study data. Third section describes the methods of measuring population projection quality. Fourth section presents the results of this study. Fifth section provides a discussion on the study results. Sixth section summarizes this study.

## Data

### Population projection datasets

In this study, we collected nine population projection datasets of China published by different scholars and organizations. The details of these population projection datasets are summarized in Table 1. We named them according to publishers' institutions or organizations' abbreviations. Additionally, China's actual population at the country and province scale from 2010 to 2020 is derived from China's Statistical Yearbook. In general, these datasets are different in spatial, temporal, and scenarios dimensions.

**Table 1:** Population data source.

No	Name	Publish year	Spatial resolution	Temporal resolution	Scenario	Publisher
1	THU	2020	30 m	2010–2100, by 1	SSP1, SSP2, SSP3, SSP4, SSP5	Tsinghua University^22^
2	NUIST	2019	0.5°	2010–2100, by 1	SSP1, SSP2, SSP3, SSP4, SSP5	Nanjing University of Information Science and Technology^21^
3	NIES	2017	0.5°	1980–2100, by 10	SSP1, SSP2, SSP3	Japanese National Institute for Environmental Studies^20^
4	SEDAC	2020	1 km	2010–2100, by 10	SSP1, SSP2, SSP3, SSP4, SSP5	Socioeconomic Data and Applications Center^19^
5	IIASA	2017	Country	2010–2100, by 5	SSP1, SSP2, SSP3, SSP4, SSP5	Institute for Applied Systems Analysis^14^
6	IHME	2020	Country	1950–2100, by 1	Reference, Slower, Faster, Fastest (female educational attainment)	Institute for Health Metrics and Evaluation^35^
7	CEPAM	2019	country	2015–2100, by 10	SSP1-Rapid Development SSP2-CEPAM Medium SSP3-Stalled Development SSP2-CEPAM Double Migration SSP2-CEPAM Zero Migration	Centre of Expertise on Population and Migration^36^
8	WCDE	2018	Country	1950–2100, by 5	SSP1-Rapid Development SSP2-Medium SSP3-Stalled Development SSP2-Medium Zero Migration SSP2-Medium Double Migrations	Wittgenstein Centre Data Explorer^37^
9	UN	2019	Country	1950–2100, by 1	Estimates Low fertility Medium fertility High fertility Instant-replacement-fertility Momentum Constant-mortality No change Zero-migration	United Nations Population Division^38^

For the spatial resolution, four datasets provide spatially explicit population distribution, including THU, NUIST, NIES, and SEDAC. Another five datasets only project total population change at the national scale. The most detailed spatial resolution is 30 × 30 m of THU.

For the temporal resolution, these datasets are different in the initial year, end years, and interval timespans. For example, five datasets provide the population from 2010 to 2100, including THU, NUIST, SEDAC, IIASA, CEPAM. In addition, the NIES, IHME, WCDE, and UN provide the estimated population in history years before 2010. As for the time interval, the THU, NUIST, IHME, and UN provide yearly population data. The IIASA and WCDE provide the population with 5 years intervals. The NIES, SEDAC, and CEPAM merely offer 10 years' interval population projection.

For the scenarios, except the IHME and UN, all datasets follow the narratives of the SSPs scenarios. SSP1 is a sustainability scenario, representing that the increase in educational level leads to low fertility in future population growth. SSP2 is the Business-as-Usual or moderate scenario, which keeps the traditional development tendency in future changes. SSP3 is the regional rivalry scenario, denoting a rapidly increasing population with high fertility to ensure abundant human labor resources. In the projection of CEPAM and WCDE, they extend the SSP2 scenario by assuming different international migration rates. The IHME focus on the role of female educational attainment in population growth. Therefore, they set four scenarios to represent different situations of female educational attainment improvement. The UN provides the most complex scenarios by combining different fertility, mortality, and international migrations.

Due to the mismatches in spatial, temporal, and scenarios, it is necessary to unify them into the same scale for comparison. Limited by the spatial resolution, we could merely compare the projection with the actual population at the country and province scale. Besides, only the spatial explicit datasets could be aggregated into provincial data, such as THU, NUIST, NIES, and SEDAC. However, we only compare the projection of NUIST and THU at the province scale, because the projected intervals of NIES and SEDAC are too long as 10 years. We compare them for the years from 2010 to 2020, due to 2010 is the initial projection year of most datasets, and 2020 is the latest population census year. Furthermore, we select the medium pathway scenario of each dataset to compare, such as the SSP2 and Middle scenarios. Because the projection in the medium scenario reflects the conditional population circumstances, and it is the basis of other scenarios. Additionally, the middle pathway is the most similar to the present world's future trajectory^33,34^.

### Impact factors of projection errors

We collect thirty demographic indicators of 31 provinces of China reflecting the population information to support the regression of SEM, as Table 2 shows. The outline indicators are the most basic information to describe the population profile for a specific province, including the total population, birth rate, mortality rate, natural growth rate, and annual population growth rate. The structure information depicts the population proportion division by the age and household registration types. The sex ratio is the number of males per 100 females. Besides the total sex ratios, we obtain the sex ratios for various population groups, such as urban, rural, and births. Fertility is significant in population projection. In this class, we obtain the number of births with different types and reflect females' reproductive situations. In the migration class, the population leaving more than half a year and the population from other provinces could represent the domestic population mobility. The number of foreigners reflects the influence of transnational migration. The economic level plays a vital role in population change. In this class, we utilize the provincial average wage and unemployment rate to depict their economic standards. The governmental policy change is the crucial impact factor for population changes. We use the expenditure of maternity insurance and hospitals' quantity to reflect government attitudes to population control. In the education class, we acquire the proportion of the population with high school education or above to depict the educational level of a certain province. To eliminate the effect of data units, all impact factor values are standardized by the Z-Score transformation.

**Table 2:** Description of the demographic and socioeconomic factors (N = 31 (province), year = 2010).

Class	No.	Name	Description	Max	Min	Mean
Outline	1	Birth rate	Birth rate (%)	6.68	15.99	11.29
	2	Mortality rate	Mortality rate (%)	4.21	6.88	5.83
	3	Natural growth rate	Natural growth rate (%)	0.42	10.56	5.46
	4	Average growth rate	Annual average growth rate, 2010–2020 (%)	-0.02	0.20	0.06
	5	Population	Total population (Person)	3.00 × 10^6^	1.04 × 10^8^	4.30 × 10^7^
Structure	6	Proportion of aged 0–14	Proportion of population aged 0–14 (%)	8.61	25.22	16.75
	7	Proportion of aged 15–64	Proportion of population aged 15–64 (%)	66.21	82.68	74.74
	8	Proportion of aged 65 and above	Proportion of population aged 65 and above (%)	5.09	11.56	8.51
	9	Proportion of none-agricultural persons	Proportion of none-agricultural population (%)	14.77	61.89	31.93
	10	Proportion of ethnic minorities	Proportion of ethnic minorities population (%)	0.00	0.92	0.15
Sex	11	Total sex ratio	Total sex ratio (%)	101.52	114.52	105.71
	12	Urban sex ratio	The sex ratio of urban population (%)	99.75	117.63	104.36
	13	Rural sex ratio	The sex ratio of rural population (%)	98.75	113.33	106.12
	14	Births sex ratio	The sex ratio of births (%)	100.08	131.07	118.39
Fertility	15	Number of births	The population of newborns (person)	2.57 × 10^3^	9.50 × 10^4^	3.84 × 10^4^
	16	Number of first child	The population of newborns as the first child in family (person)	1.15 × 10^3^	5.76 × 10^4^	2.39 × 10^4^
	17	Number of second child	The population of newborns as the second child in family (person)	7.53 × 10^2^	3.44 × 10^4^	1.20 × 10^4^
	18	Number of third child	The population of newborns as the first third in family (person)	1.42 × 10^2^	6.85 × 10^3^	2.03 × 10^3^
	19	Number of childbearing women	The population of females aged from 15 to 65 (person)	7.52 × 10^5^	2.77 × 10^7^	1.04 × 10^7^
	20	Number of abortions	The population of abortions (person)	9.85 × 10^2^	1.05 × 10^6^	2.05 × 10^5^
	21	Total fertility rate	The average number of children of female (person)	0.71	1.79	1.86
	22	Contraceptive rate of married women	The rate of childbearing women take contraceptive after married (%)	77.96	93.93	88.00
Migration	23	Number of people from other provinces	The population from other provinces (person)	1.65 × 10^5^	2.15 × 10^7^	2.77 × 10^6^
	24	Number of foreigners	The population of foreigners (person)	3.79 × 10^2^	3.16 × 10^5^	3.29 × 10^4^
	25	Proportion of population leaving more than half-year	The proportion of person leaving the province more than 6 months (%)	5.31	29.72	19.44
Economic	26	Average wage	The average wage of the province (¥ Yuan)	2.77 × 10^4^	6.61 × 10^4^	3.61 × 10^4^
	27	Unemployment rate	The unemployment rate of the province (%)	1.40	4.40	3.63
Policy	28	Number of tertiary hospitals	The number of tertiary hospitals (number)	0.10	13.30	3.55
	29	Maternity insurance expenditure	The expenditure of governmental maternity insurance (billion ¥ Yuan)	0.20	8.50	4.06
Education	30	Proportion of population above high school	Proportion of population above high school	0.01	0.60	0.09

## Methods

**Figure 1:**
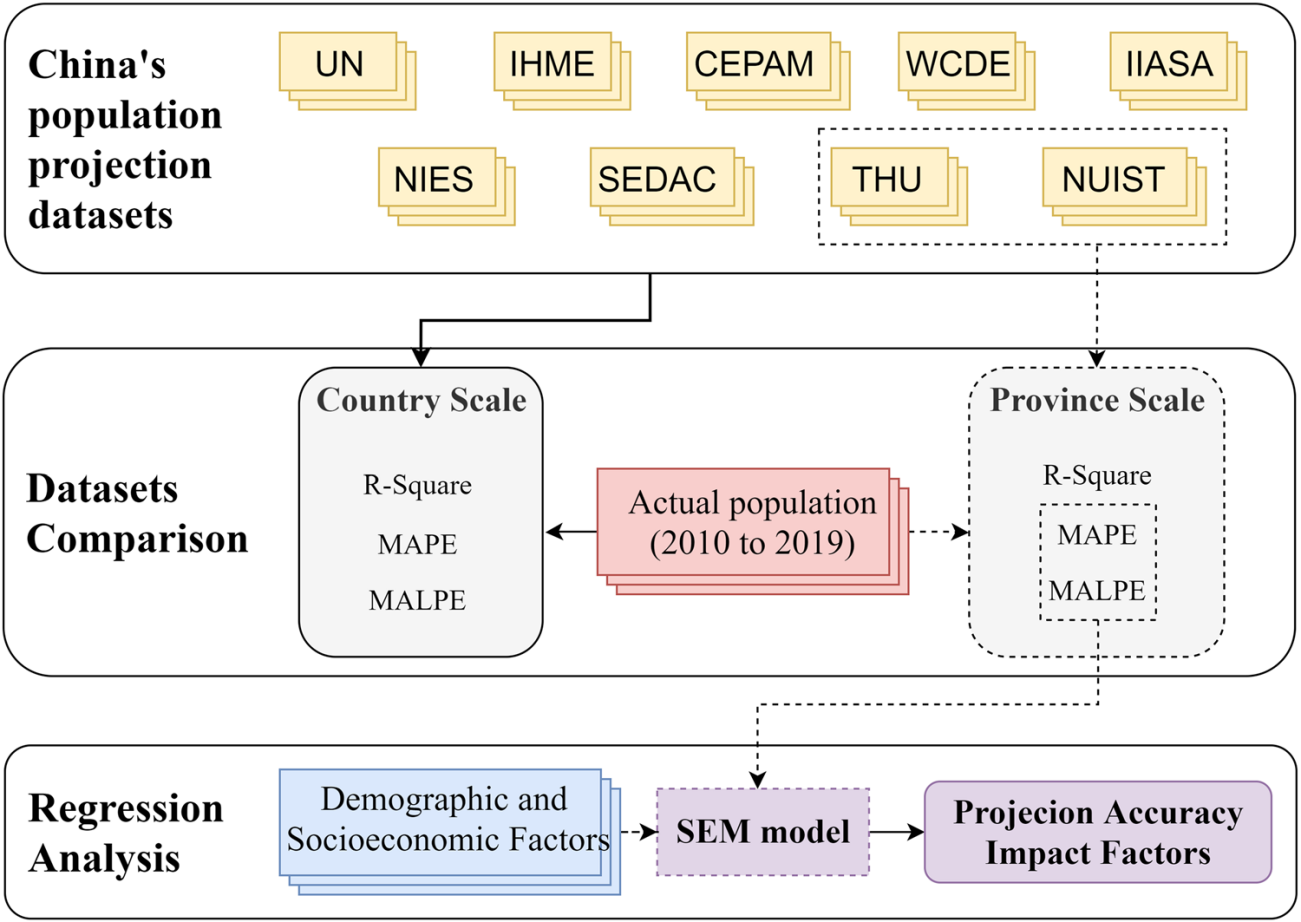
Workflow of the research.

The Fig. 1 shows the research workflow of this study. In the beginning, we collect nine projection datasets from various scholars and organizations. Then, we evaluate them with the actual population data from 2010 to 2020 at the country and province scale. Besides, we utilize the mean absolute percentage error (*MAPE*), mean algebraic percentage error (*MALPE*), and R-Square to measure the quantitative differences between actual and projection populations. Finally, we employ the SEM regression models to explore the impact factors for projection accuracy.

### Measurements of population projection errors

Generally, national official census data is the most reliable population criteria^39–41^. Smith proposed evaluating the population projection data quality by examining its projection accuracy and projection bias^42^. Projection accuracy is the absolute difference between projected and actual values, and it expresses the degree of error deviation^43,44^. Projection bias is the real difference between the values, and it shows the direction and magnitude of the projection error^32,45^. Therefore, we select the *MAPE* (Eq. (1)) and the *MALPE* (Eq. (2)) to indicate the population projection accuracy and projection bias, respectively^46^. Moreover, we utilize the coefficient of determination ${(R}^{2}$, Eq. (3)). In this study, we calculate these projection error indicators at the country and province scales. These indicators are calculated as follows:

1 $$MAPE\left(\mathrm{\%}\right)=\frac{\sum \left|\frac{{P}_{t}-{A}_{t}}{{A}_{t}}\right|}{n}\times 100$$

2 $$MALPE \left(\mathrm{\%}\right)=\frac{\sum \left(\frac{{P}_{t}-{A}_{t}}{{A}_{t}}\right)}{n}\times 100$$

3 $${R}^{2}=1-\frac{\sum {({P}_{t}-{A}_{t})}^{2}}{\sum {({P}_{t}-\overline{A })}^{2}}$$

In the above equations, $t$ is a year from 2010 to 2020, $n$ is 11 years. $P$ is the population projection datasets, $A$ is the actual population data. ${P}_{t}$ and ${A}_{t}$ is the projected and actual population in the $t$ year. According to the equations, a positive *MALPE* indicates that the projection is greater than the actual values and a negative *MALPE* means that the population projection is less than the actual values. The *MAPE* is a nonnegative value without upper limitations. The zero *MAPE* indicates that the projection results are entirely correct, and a large *MAPE* indicates lower projection accuracy. The percentage variables are unit-free and easy to understand and interpret. Thus, they are standard measurements in the applied demography literature ^31,47^.

### Attribution analysis of projection errors

We utilize spatial error regression models to analyze the possible relationships between the projection accuracy and those impact factors. The SEM could discover the spatial autocorrelation of variables, allowing us to explore deeper spatial associations that the ordinary linear regression model cannot reveal^48,49^. Due to there are only eleven years intervals of validation data as samples, it could not support the attribution analysis at the country scale. Therefore, we merely analyze the impact factors of population projection at the province level. As a result, the dependent variables of the SEM models are the *MAPE* and *MALPE* of China's 31 provinces from 2010 to 2020. The explanatory variables are the provincial demographic and social indicators, as Table 2 shown.

In the SEM, mutual effects are assumed for neighboring districts' same explanatory variables, and the dependent variables have no spatial correlations. Therefore, the formulas of SEM are shown as Eq. 4 and Eq. 5. $Y$ is the $n\times 1$ vector of response variables, $X$ is an $n\times p$ matrix of the explanatory variable, $\beta$ is an $p\times 1$ vector of regression coefficients, $\varepsilon$ represent "white noise," $u$ is the error refers to spatial variations, ${W}_{1}$ is the spatial weight matrix describing the spatial mode of residuals, and $\lambda$ is the parameter of the spatial error term. The closer $\lambda$ is to 1, the more similar the explanatory variables in neighboring places.

4 $$Y=X\beta +u$$

5 $$u={\lambda W}_{1}+\varepsilon$$

In this study, the dependent variables are the *MAPE* and *MALPE* of China's 31 provinces; thus, the SEM model's $Y$ matrix is a $31\times 1$ vector. We utilize the stepwise method to select the explanatory variables when constructing the SEM model to handle multicollinearity among variables. Therefore, the final models could contain different impact factors.

## Results

### Country scale comparison

**Figure 2:**
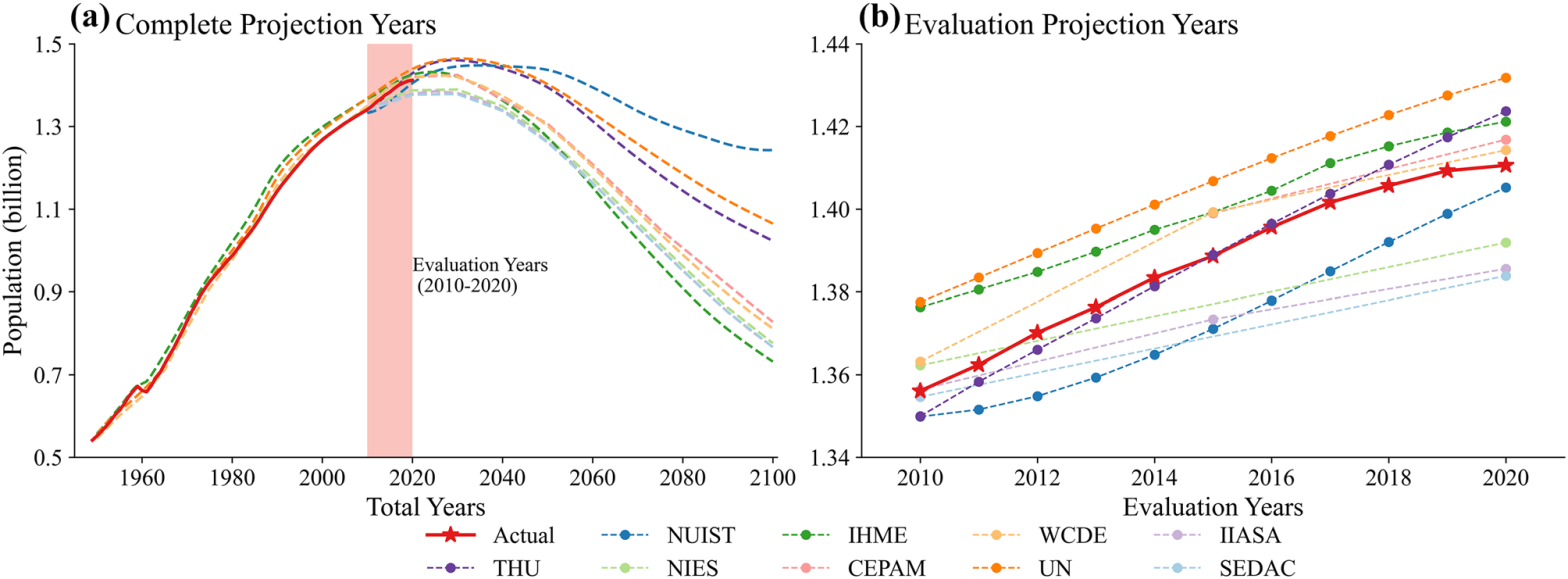
Comparison between the actual and projection population at the country scale. The vertical axis is the population number (unit: billion), and the horizontal axis represents the years.

As Fig. 2a shows, all dataset projection population to 2100, but four datasets provide population start before 2010, and other five start from 2010. The IHME, UN, and WCDE are higher than the actual data since the 1970s, yet the WCDE coincides with the proper condition in most historical years.

In the evaluation years from 2010 to 2020 (Fig. 2b), most projections show an approximatively linear growth trend, and they do not foresee the inflection point arising prematurely in 2017. Only the WCDE reveals the slowdown tendency from 2015 to 2020. In this period, the projections of UN, IHME, WCDE, and CEPAM are higher than the actual population. The NIES, IIASA, SEDAC, and NUIST are lower than the truth, but the NUIST approaches the actual value gradually from 2015. The projection of THU is the closest to the actual population curve from 2010 to 2017, but it overestimates the population after 2017 as well.

According to the long-term population projection results, China's population size is generally projected to peak and show a decreasing trend shortly. These datasets predict China's maximum population is between 1.38 billion to 1.45 billion (Supplement Table S1). The IHME thinks the population will reach the peak in 2024 as the fastest growth among projections. The NUIST considers it will be maximum in 2034 as the latest. The average value for the maximum population year of all projections is 2028. Furthermore, these datasets show three types of trajectories after reaching the population peak. The NUIST reveals the most slowly population decreasing trend, and it thinks the population will be 1.24 billion in 2100, which is the highest in nine datasets. The UN and THU represent the medium population reduction situations, and they project the population will maintain 1 billion above in 2100. The rest six datasets show the total population will sharply decrease under 0.9 billion in 2100.

The quantitative indicators for measuring projection accuracy and bias during the validation years are calculated in Table 3. The THU has the lowest *MAPE* and *MALPE*, and the largest ${R}^{2}$ as 41.40%, 8.00%, and 0.90 respectively, thus it is the best projection dataset in this period. Inversely, the projection of UN is the worst among these datasets.

**Table 3:** Measurements of projection error from 2010 to 2020.

	MAPE (%)	MALPE (%)	${\mathrm{R}}^{2}$
THU	41.40	8.00	0.90
NUIST	127.88	− 127.88	0.38
NIES	115.93	− 56.11	0.74
IHME	117.15	117.15	0.50
CEPAM	77.34	77.34	0.39
WCDE	67.51	67.51	0.88
UN	176.28	176.28	− 0.06
IIASA	125.72	− 123.61	0.43
SEDAC	130.09	− 130.09	0.52

Furthermore, we could reveal the direction of projection bias by analyzing the relation between *MAPE* and *MALPE*. For example, THU's *MALPE* is lower than *MAPE* significantly, which reveals both overestimate and underestimate for THU, but the overestimates cause more projection accuracy loss. The WCDE takes the second high projection accuracy with equal *MAPE* and *MALPE*. Thus it overestimates the population for each year in this period. In summary, the projection accuracy loss of NUIST, NIES, IIASA, and SEDAC is caused by the negative errors, and the THU, IHME, CEPAM, WCDE, and UN are own to positive errors.

### Province scale comparison

**Figure 3:**
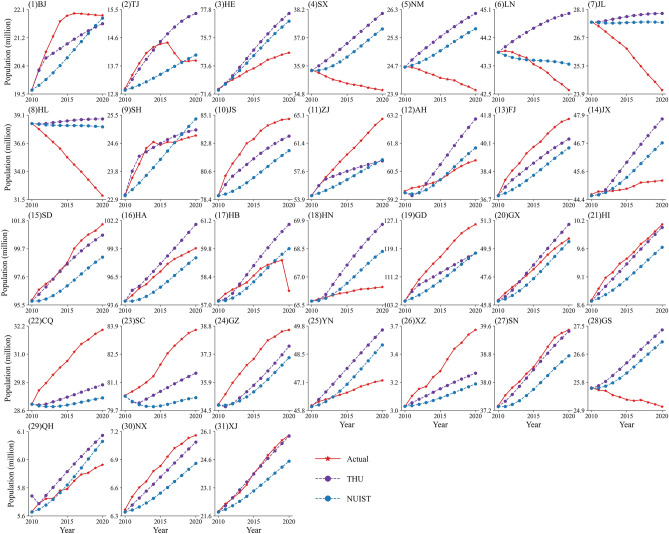
Comparison between actual and projection population at the province scale. The subfigures from (1) to (31) represent different provinces. The vertical axis is the population number (unit: million), and the horizontal axis represents the validation years from 2010 to 2020. The red star indicates the actual provincial population, the blue circle represents the NUIST, and the purple circle denotes the THU.

At the province scale, the THU and NUIST are validated with each actual provincial population, and the results are shown in Fig. 3. The results reveal that NUIST and THU have various conditions in different provinces with over or underestimated projection compared to the actual population.

The first pattern is that the actual population turns from rapid to slow growth, such as Beijing, Tianjin, and Shanghai (Fig. 3 (1, 2, 9)). In these provinces, the THU discovers the slowdown trend of population, but NUIST keeps linearly growing without turning points in the period.

The second pattern is that the actual population keeps reducing in the period, but both projections show population increasing (Fig. 3 (4, 5, 6, 7, 8, 28)). The pattern includes six provinces as Shanxi, Neimenggu, Liaoning, Heilongjiang, Jilin, and Gansu provinces. These provinces are all located in the northeast and northwest of China, and they have experienced severe population loss in the last years. However, the two projections do not expect such a rapid population reduction in these regions.

The third pattern is that the actual population remains to increase, but both THU and NUIST overestimate the tendency. There are six provinces in this category, containing Hebei, Anhui, Jiangxi, Hunan, Yunnan, and Qinghai provinces (Fig. 3 (3, 12, 14, 18, 25, 29)).

The fourth pattern is that the THU and NUIST underestimate the actual growth population. This class includes thirteen provinces as Jiangsu, Zhejiang, Fujian, Shandong, Guangdong, Hainan, Chongqing, Sichuan, Guizhou, Xizang, Shannxi, Ningxia, and Xinjiang province. (Fig. 3 (10, 11, 13, 15, 19, 21, 22, 23, 24, 26, 27, 30, 31)). These provinces are primarily located in the southwest and southeast coastal areas, revealing that the population of south China is maintaining increasement.

The fifth pattern is that the actual population is less than THU but larger than NUIST. The three provinces as Hunan, Hubei, and Guangxi belonging to the type. In this type, both the two projections are closed to the truth.

**Figure 4:**
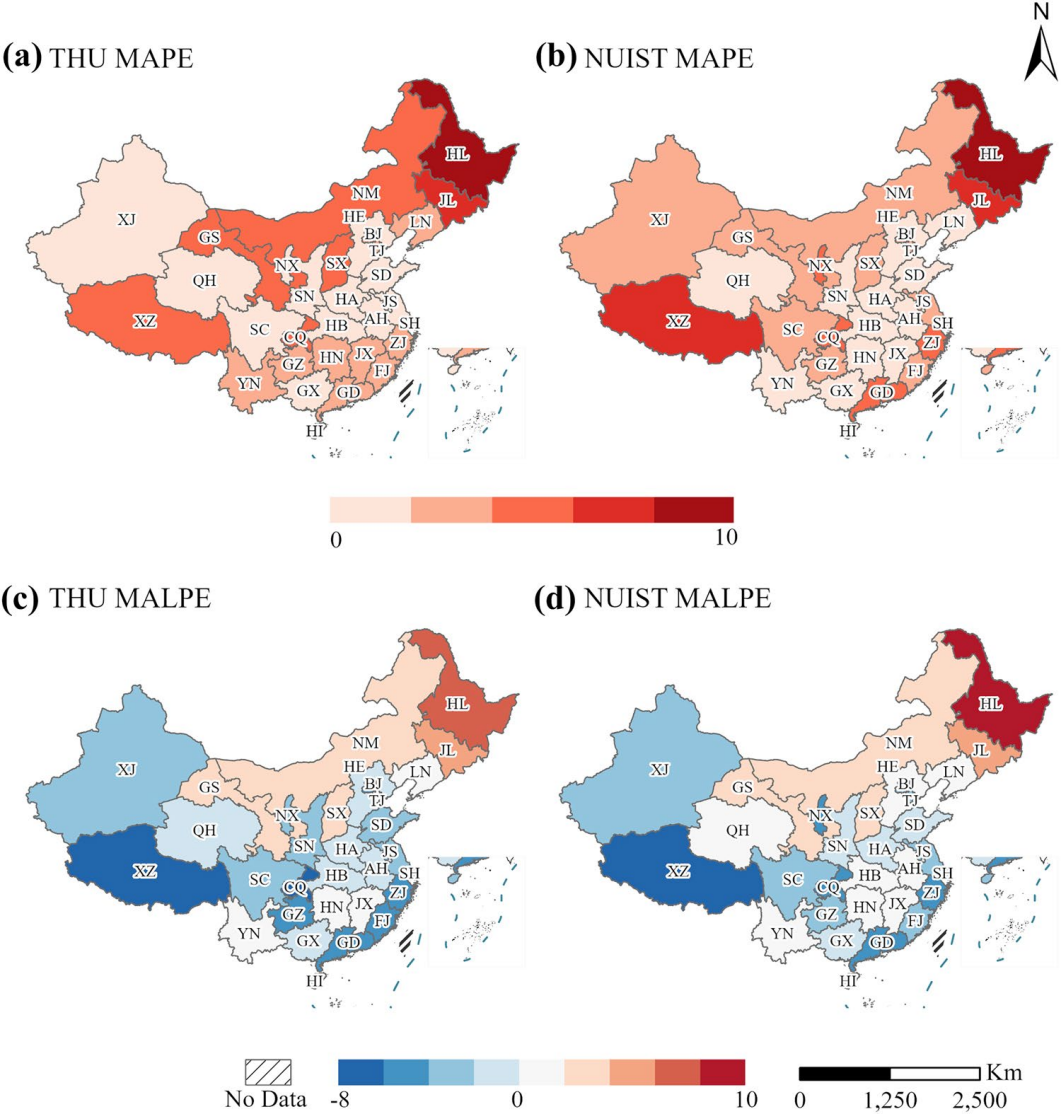
Distribution of the provincial *MAPE* and *MALPE* of NUIST and THU. The left panel (**a**, **c**) shows the THU results, and the right panel (**b**, **d**) shows the NUIST results.

We utilize the *MAPE* and *MALPE* to measure the projection errors at the province scale quantitatively, and the results are displayed in Fig. 4. There are differences in the two datasets' *MAPE* (Fig. 4a, b). THU's *MAPE* distribution could be divided into three distinct portions from south to north China, the center regions have the least values, and the northeast provinces own the largest values. Moreover, the NUIST's *MAPE* distributions display three sections from east to west China, the northeast and southeast provinces have the highest values, and the middle region has the lowest values. As a result, both THU and NUIST have large errors in northeast China and Xizang province.

For the *MALPE*, the THU and NUIST have similar spatial distributions as the Fig. 4c, d shown. The red color indicates the projection overate the population, and the blue color means the projection underestimates the population growth in a certain province. Therefore, both the THU and NUIST overestimate the population development in north China, especially the northeast regions such as Heilongjiang, Jilin, and Neimenggu province. The overestimated projection may be caused by they do not consider the population outflow in these areas. Besides, the southeast coastal provinces and southwest provinces own the negative *MALPE*, denoting their population are underestimated.

**Figure 5:**
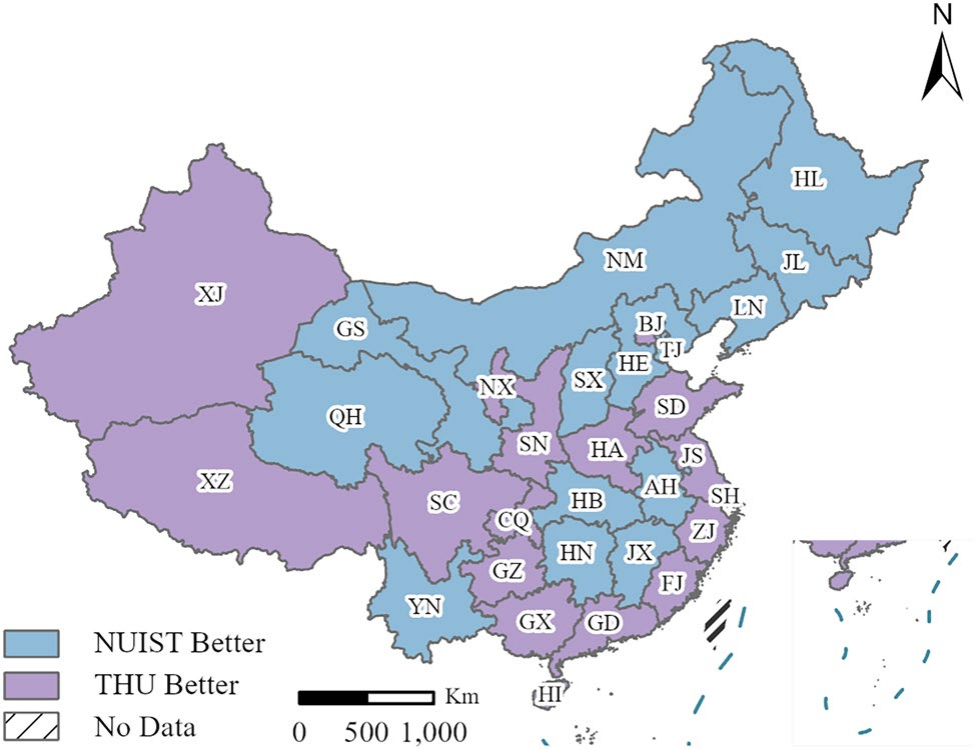
Projection accuracy comparison of THU and NUIST. The purple color indicates that THU has a lower *MAPE* than NUIST, and the blue color indicates that NUIST has a lower *MAPE* than THU in a particular province.

Additionally, we compare the NUIST and THU's projection accuracies from 2010 to 2020 based on their *MAPE* values. We calculate the difference for the *MAPE* of NUIST and THU. When the difference is positive, the NUIST projection is more inaccurate than the THU projection. In contrast, if the difference is negative, the NUIST projection is more accurate than the THU projection in the individual province. The *MAPE* comparison results are displayed in Fig. 5.

The purple color indicates that the THU projected population is more accurate than the NUIST projected population. Conversely, the blue indicates that the NUIST projected population is closer to the actual population than the THU population. According to the Fig. 5, the purple regions are primarily distributed in the western and northern coast of China, such as Xingjiang, Xizang, Guangdong, and Shangdong province. The blue regions are mainly located in the northeastern and central of China, such as Heilongjiang, Jilin, Hubei, and Jiangxi province.

### Attribution of the population projection error

**Figure 6:**
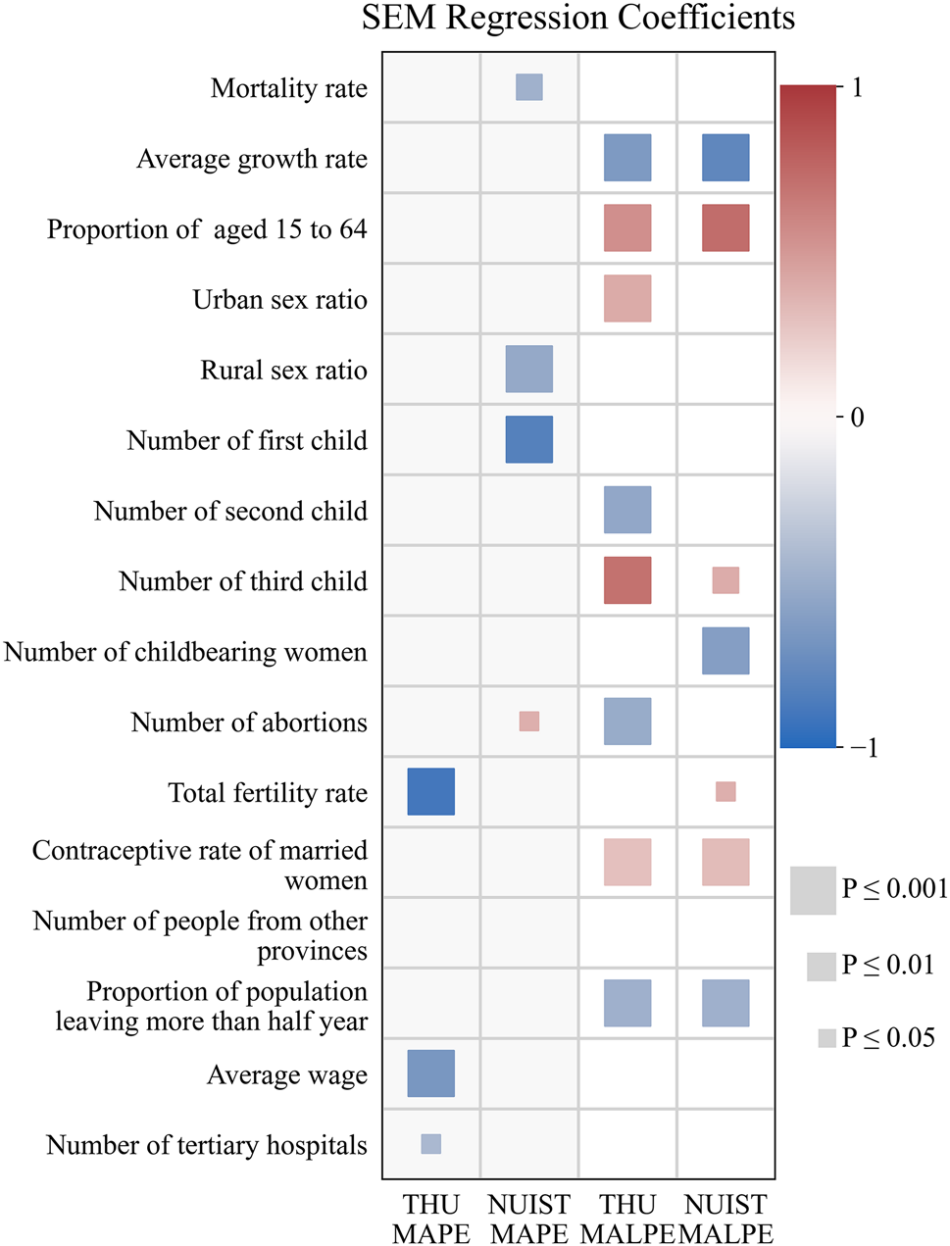
Regression coefficients of the SEM for the *MAPE* and *MALPE* of NUIST and THU.

We utilize the SEM model to analyze the impact factors of projection errors at the province scale. Therefore, there are four models for *MAPE* and *MALPE* of THU and NUIST, and their regression coefficients are displayed in Fig. 6. In this figure, only the impact factors with significant influence are drawn. The square size represents the significance level of impact factors, and the color indicates the regression coefficient value.

Three impact factors significantly influence THU's *MAPE*, including the total fertility rate, average wage, and the number of tertiary hospitals. Besides, all the indicators are negatively related to the THU's *MAPE*, which means the provinces with lower fertility rates, average wages, and more tertiary hospitals would have worse projection results. For instance, in the northeast provinces with low fertility rates, the THU's projection errors are higher than NUIST. Furthermore, four impact factors are related to NUIST's *MAPE*, including the mortality rate, rural sex ratio, number of the first child, and number of abortions.

When analyzing the impact factors of *MALPE*, it is necessary to consider the positive or negative of values. As shown in Fig. 5, there are five provinces with the positive *MALPE*, including the Heilongjiang, Jinlin, Neimenggu, Shanxi, and Gansu provinces. According to the Fig. 6, the *MALPE* of THU and NIUST are influenced by some common impact factors. For example, the proportion of the population aged 15 to 64, the contraceptive rate of married women, and the number of third children have a significantly positive relation with *MALPE*. Therefore, for the provinces with negative *MALPE*, the provinces with more population aged 15 to 64 would have higher projection accuracy.

On the contrary, the average population growth rate and proportion of the population leaving the province for more than a half year negatively correlate with *MALPE*. Therefore, their negative population growth rates expand projection errors for the provinces with positive *MALPE*, such as the Heilongjiang and Jilin provinces. Similarly, the provinces with negative *MALPE* and positive population growth rates face more significant projection errors, such as the Guangdong and Zhejiang province. Meanwhile, the proportion of the population leaving more than half a year is negatively related to the *MALPE*. As a result, the more significant growth rate and population migration lead to higher projection errors for the two datasets.

## Discussion

### The deceleration of China's population growth rate

The slowing of China's total population development starts from 2017 (Fig. 2b), yet some provinces' population reduction from 2010 already (Fig. 3). However, these projections datasets do not anticipate the turning point of China's population growth coming so early and population reduction so sharply for some provinces.

The overestimate of total fertility rate (TFR) in projection is a significant reason for overrating the population growth. Due to many studies deem the TFR 1.180 in the sixth national population census is severely underestimated^50,51^, the TFR in 2010 is rectified higher in all projections, as Table 4 shows. The UN offers the maximum TFR of 1.620, and IHME provides the minimum TFR of 1.220. The THU and NUIST up-regulate the projected TFR of 2020 and 2030, because they think the loosened governmental birth control policies will facilitate birth effectively. Nevertheless, according to the newest seventh national population census, the TFR is merely 1.300 in 2020. Therefore, the IHME, CEPAM, and WCDE are approaching the actual population because their TFR is closer to the census results. The UN and THU are higher than the actual condition in 2020 seriously.

**Table 4:** The total fertility rate (TFR) and net migration of projection datasets.

	Total fertility rate (‰)									
	2010	2020	2030	2040	2050	2060	2070	2080	2090	2100
Actual	1.180	1.300								
THU	1.600	1.800	1.650	1.706	1.706	1.706	1.706	1.706	1.706	1.802
NUIST	1.450	1.690	1.720	1.690	1.660	1.660	1.660	1.640	1.640	1.640
IHME	1.220	1.455	1.421	1.431	1.441	1.452	1.457	1.457	1.460	1.466
CEPAM		1.470	1.420	1.390	1.390	1.410	1.430	1.460	1.460	1.510
WCDE	1.580	1.440	1.370	1.370	1.400	1.410	1.430	1.450	1.470	1.490
UN	1.620	1.690	1.720	1.730	1.750	1.760	1.760	1.770	1.770	1.770
IIASA/NIES/SEDAC	1.500	1.400	1.400	1.400	1.400	1.400	1.400	1.500	1.500	1.500

	International net migration (person)									
	2010	2020	2030	2040	2050	2060	2070	2080	2090	2100
THU	−401,881				−401,881					0
NUIST	−475,859									−191,410
IHME		−470,447	−495,422	−430,100	−279,577	−122,471	75,908	238,645	354,147	358,804
CEPAM		−918,100						−580,400		−453,600
WCDE	−478,180	−183,700	−183,780	−175,580	−162,940	−147,580	−130,780	−114,680	−100,680	−88,900
UN	−435,600	−348,400	−352,200	−311,800	−310,000	−310,000	−310,000	−310,000	−310,000	−310,000
IIASA/NIES/SEDAC	−377,800	−377,800	−377,800	−377,800	−377,800	−377,800				0

*The empty table cells mean original researches do not provide these data.

After publishing the low TFR in the seventh census results, the worries for maintaining China's future population steadily growth are discussed again. For China, the present TFR is lower than the replacement-level fertility, which means the new generations will be seriously less than the aged population in the future. The Sub-replacement fertility probably leads to the labor shortage, economic contraction, and increased social pensions burden^52^. Therefore, China's government implemented the "Three-Child" policy allowing couples to nurse three children in 2020 after the "Two-Child" policy permitting two children in for family in 2015^53^. However, considering the actual TFR does not realize the high level as THU, NUIST, and UN projected in the validation years, the population may reach a peak earlier than these datasets projected. Besides, to avoid the population decline as the IHME, IIASA and WCDE predicted, China's fertility regulation implements may need further loosened.

On the other hand, international net migration has an import effect on China's future population change. As Table 4 shows, the net migration values are assumed unchanged for a time in some projections. For example, the UN supposes the migration invariability from 2040 to 2100, and the IIASA assumes it unchanged from 2010 to 2060. Moreover, in the projection of the THU and IIASA, they believe the net migration would gradually equal to zero in 2100, as Abel stated^54^. Nevertheless, other projections do not set the net migration as zero in 2100. Furthermore, although UN keeps a high TFR in the total periods, its projection population is not the largest, which could be attributed to its large population outflow. Similarly, the IHME supposed population inflow would be since 2070, but its low fertility hypothesis predicts the lowest population in 2100. As a result, the migration should be set based on more reasonable methods.

### The imbalance of population growth in north–south China

As shown in Fig. 4c, d, the projections cannot reflect the radical population reduction in northeast provinces and underrate the increase in southwest and southeast provinces. For example, the THU thinks their population keeps increasing in Liaoning, Jilin, and Heilongjiang provinces with linear population decrease, and THU predicts it decrease with gentle rates. The unpredictable population reduction may be ascribed to these models underrate the population outflow intensity in northeast China. Besides, the population reduction is caused by low fertility and influenced by economic and social factors. Moreover, in southeast China such as Guangdong and Zhejiang province, both projections are lower than the actual values, which may be caused by their flourishing economic activities attracting plenty of population inflow^55^.

In southwest China, the projections seriously underrate the population of Chongqing, Sichuan, and Xizang. These errors are likely because the government policies boost economic development and attract more population inflow. For instance, the "Cheng-Yu Economic Zone" policy was introduced in 2011, which accelerated the economic development and population expansion of Chongqing and Sichuan Provinces^56,57^. Due to China's "poverty alleviation" policies, Xizang has received generous economic assistance from the central government to support its rapid development^58^. However, the population projection models are unable to consider the policy changes.

### Factors that impact the projection accuracy

According to the SEM regression results, some common factors impact the projection accuracy for THU and NUIST. The first category is the population change indicators, as the population growth rates and province-cross migration persons. The high population annually change rate extends the projection errors, and the population migration also brings excellent uncertainty to projections. Due to the siphonage phenomenon, the urban agglomeration regions constantly attract populations from other undeveloped provinces, such as the Pearl River Delta and Yangtze River Delta regions^59,60^. However, the population projection models of THU and NUIST oversimplified the depiction of migration internal China. As a result, their projections overestimated population outflow provinces and underestimated provinces with massive population inflow.

Moreover, the proportion of the population aged 15 to 64 also significantly impacts the projection accuracy. Based on the regression results, the more population in this group, the higher the projection accuracy. Because the group accounts for the largest in the total population, and it is also the primary fertility group. If the projection could not acquire reasonable population and fertility rates in the group, the entire total population projection may be seriously inaccurate.

Furthermore, the contraceptive rate of childbearing married women significantly influences projection accuracy, and the indicator could denote people's fertility desire. Generally, the population projection models estimate the future population change based on fertility, mortality, and migration rates. However, these general parameters are challenging to represent individual s' mentality thoughts. Besides, society, economy, and culture play an essential role in people's fertility desire. Therefore, the accurate population projection needs reasonable parameters of fertility, mortality, and migrations. However, fertility is depended on the individual's choice and very personal behavior. While building the population projection models, scholars should combine the influence of society and the environment.

## Conclusion

In this study, we evaluate the projection accuracy of some population projection datasets of China. Nine datasets are compared with the actual population from 2010 to 2020 at the country scale. The projections of THU and NUIST are validated at the province scale in the same periods. Besides, we utilize the *MAPE*, *MALPE*, and R-Square to quantificationally measure the projection errors. Furthermore, we analyze the contributions of several impact factors to the projection errors based on SEM regression models. According to study results, these projections provide various population growth situations at the country and province scale, but most of them cannot show the deceleration of population growth after 2017. Moreover, the annual population change rates and the migration population significantly influence the projection accuracy. Finally, we discuss the different fertility values between the actual condition and projection set and provide suggestions for further population projection models.

## Supplementary Information

Supplementary Table 1.

## Data Availability

The actual demographical data are available from the National Bureau of Statistics of the People's Republic of China (http://www.stats.gov.cn/tjsj/pcsj/rkpc/6rp/indexch.htm). The population projection data of Nanjing University of Information Science and Technology (NUIST) are available from the website at https://geography.nuist.edu.cn/2019/1113/c1954a147560/page.htm. The population projection data of Tsinghua University (THU) are available from the website at https://doi.org/10.6084/m9.figshare.c.4605713. The population projection data of the International Institute for Applied Systems Analysis (IIASA) are provided on the websites of the SSP database (https://tntcat.iiasa.ac.at). The population projection data of the United Nations are available from the website at https://population.un.org/wpp/. The National Institute for Environmental Studies (NIES) population projection data are available from the website at https://www.cger.nies.go.jp/gcp/population-and-gdp.html. The population of Socioeconomic Data and Applications Center (SEDAC) are derived from https://sedac.ciesin.columbia.edu/data/set/popdynamics-1-km-downscaled-pop-base-year-projection-ssp-2000-2100-rev01. The data of the Institute for Health Metrics and Evaluation (IHME) is downloaded from http://ghdx.healthdata.org/record/ihme-data/global-population-forecasts-2017-2100. The data of the Centre of Expertise on Population and Migration (CEPAM) are acquired from https://core.ac.uk/display/158646554?source=2. The data of Wittgenstein Centre Data Explorer (WCDE) are obtained from http://dataexplorer.wittgensteincentre.org/wcde-v2.
