# Supplementary material for: Evaluation and analysis of the projected population of China
Source: Sci Rep. 2022 Mar 7;12:3644. doi: 10.1038/s41598-022-07646-x (PMC8901741; doi:10.1038/s41598-022-07646-x)
Supplement: Supplementary file 1 — Supplementary Table 1. [file 41598_2022_7646_MOESM1_ESM.docx]

**Table S1:** The China's maximum population of different datasets projected.

|  | **THU** | **NUIST** | **NIES** | **IHME** | **CEPAM** | **WCDE** | **UN** | **IIASA** | **SEDAC** |
| --- | --- | --- | --- | --- | --- | --- | --- | --- | --- |
| **Year** | 2029 | 2034 | 2030 | 2024 | 2030 | 2025 | 2031 | 2025 | 2030 |
| **Population**  **(billion)** | 1.46 | 1.45 | 1.39 | 1.43 | 1.42 | 1.43 | 1.46 | 1.39 | 1.38 |
